# Supplementary material for: The interactome of intact mitochondria by cross-linking mass spectrometry provides evidence for coexisting respiratory supercomplexes
Source: Mol Cell Proteomics. 2017 Dec 8;17(2):216–32. doi: 10.1074/mcp.RA117.000470 (PMC5795388; doi:10.1074/mcp.RA117.000470)
Supplement: Supplemental Data [file supp_RA117.000470_133922_0_supp_23978_fzffwf.zip › spectra_annotation/mito_spectra_annotation/271-1-3-1-5-1.pdf]

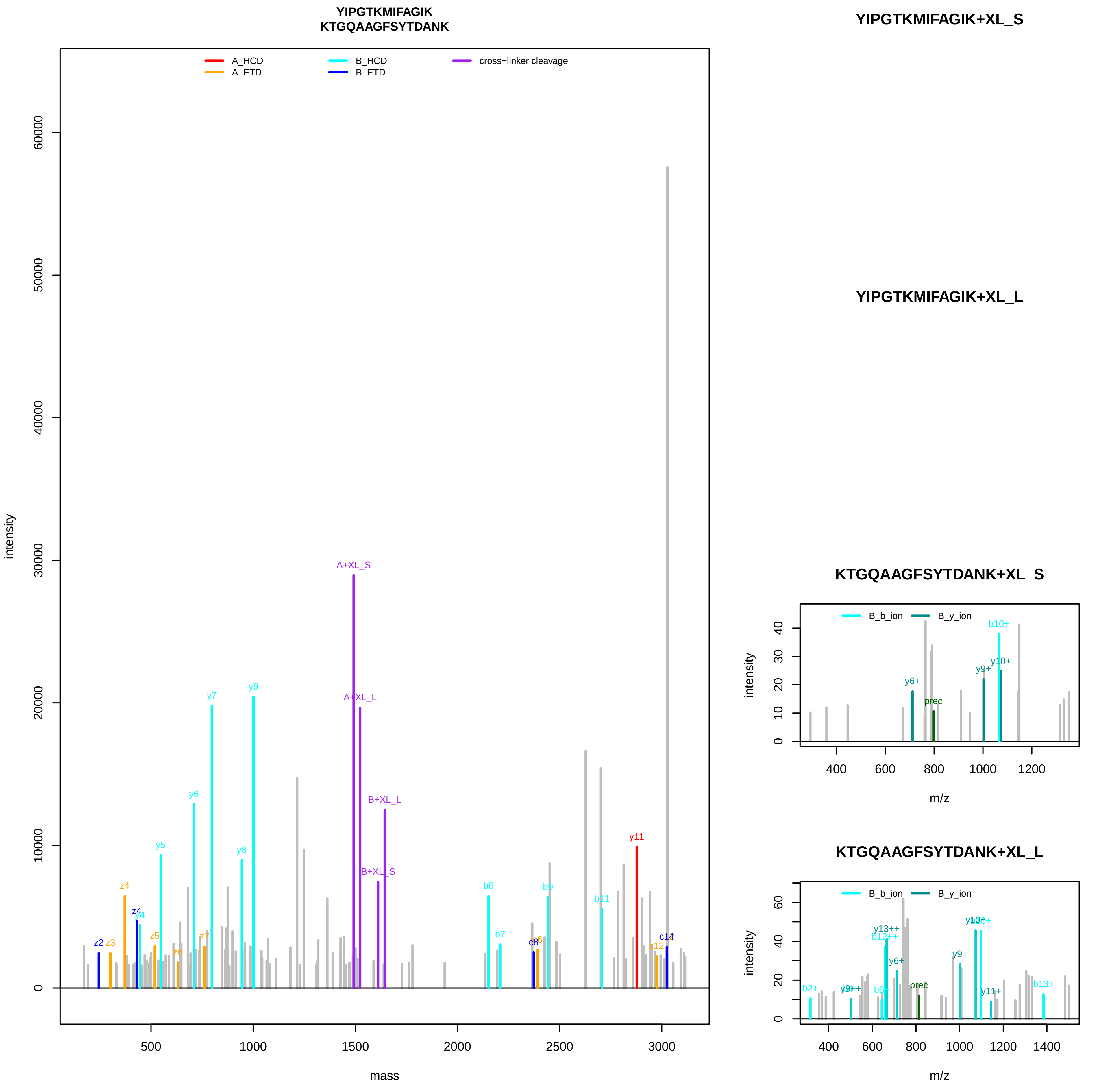
YIPGTKMIFAGIK
KTGQAAGFSYTDANK
A_HCD
A_ETD
B_HCD
B_ETD
cross−linker cleavage
intensity
mass
A+XL_S
A+XL_L
B+XL_L
B+XL_S
y11
c14
c8
b6
b7
b9
b11
y4
y5
y6
y7
y8
y9
z2
z3
z4
z5
z6
z7
z12
YIPGTKMIFAGIK+XL_S
YIPGTKMIFAGIK+XL_L
KTGQAAGFSYTDANK+XL_S
B_b_ion
B_y_ion
b10+
y10+
y9+
y6+
prec
m/z
KTGQAAGFSYTDANK+XL_L
y13++
b12++
y11+
b13+
b2+
b6+
